# Author Correction: In situ X-ray and acoustic observations of deep seismic faulting upon phase transitions in olivine

**DOI:** 10.1038/s41467-024-45682-5

**Published:** 2024-02-09

**Authors:** Tomohiro Ohuchi, Yuji Higo, Yoshinori Tange, Takeshi Sakai, Kohei Matsuda, Tetsuo Irifune

**Affiliations:** 1 Geodynamics Research Center, Ehime University, Matsuyama, 790-8577 Japan; 2 Japan Synchrotron Radiation Research Institute, Sayo, Hyogo 679-5198 Japan; 3 Sumitomo Electric Industries Ltd., Itami, Osaka, 664-0016 Japan; 4 Earth-Life Science Institute, Tokyo Institute of Technology, Tokyo, 152-8550 Japan

**Keywords:** Mineralogy, Petrology, Seismology

Correction to: *Nature Communications* 10.1038/s41467-022-32923-8, published online 15 September 2022

The original version of the Supplementary Information contained an error in Supplementary Table 1, in which the value of Hgb* was incorrectly listed as “502 kJ/mol” where it should have been “270 kJ/mol”. The HTML has been updated to include a corrected version of the Supplementary Information.

### Supplementary information

Supplementary Information

