# Supplementary material for: In situ X-ray and acoustic observations of deep seismic faulting upon phase transitions in olivine
Source: Nat Commun. 2022 Sep 15;13:5213. doi: 10.1038/s41467-022-32923-8 (PMC9477848; doi:10.1038/s41467-022-32923-8)
Supplement: Supplementary file 1 — Supplementary Information [file 41467_2022_32923_MOESM1_ESM.pdf]

Supplementary Information to "In situ X-ray and acoustic observations of deep seismic faulting upon phase transitions in olivine"

T. Ohuchi, Y. Higo, Y. Tange, T. Sakai, K. Matsuda and T. Irifune

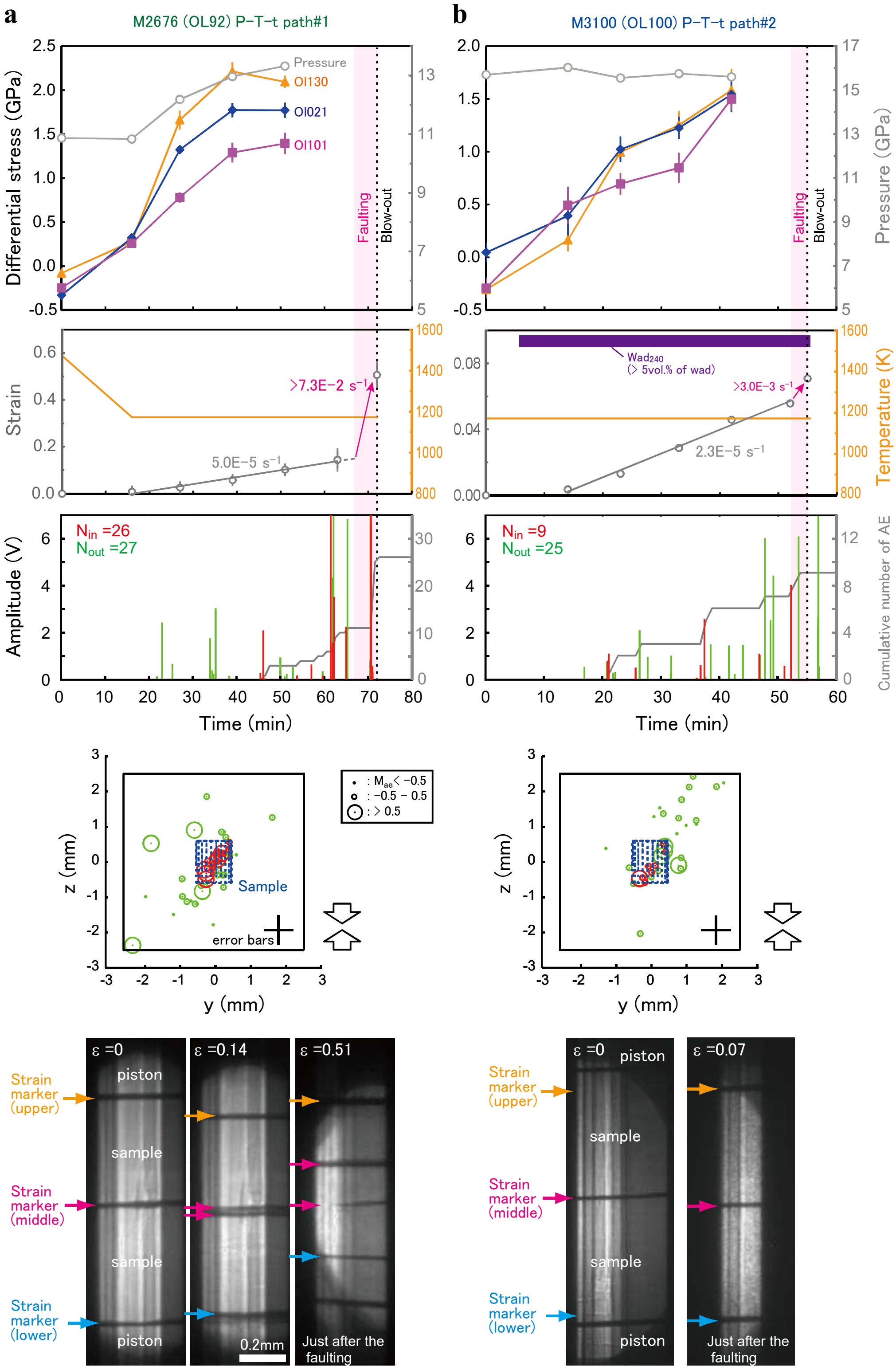

Supplementary Figure 1 | Mechanical (pressure, stress, and strain) and acoustic records plotted against time during the deformation stage in two runs in which throughgoing faulting occurred. (**a**) M2676. (**b**) M3100. Stress values were obtained from three diffraction peaks of olivine (solid diamonds: 021, solid squares: 101, solid triangles: 130). Duration in which wadsleyite 240 (Wad_{240}) peak was detected is highlighted by the thick purple line. Other symbols, lines and error bars have the same meanings as shown in Fig. 3. X-ray radiographs of the deforming samples are also shown. Splitting of the middle strain marker is observed at a strain ε =0.14 (at 62 min) in run M2676. Direction of the incident X-ray is perpendicular to the radiograph images.

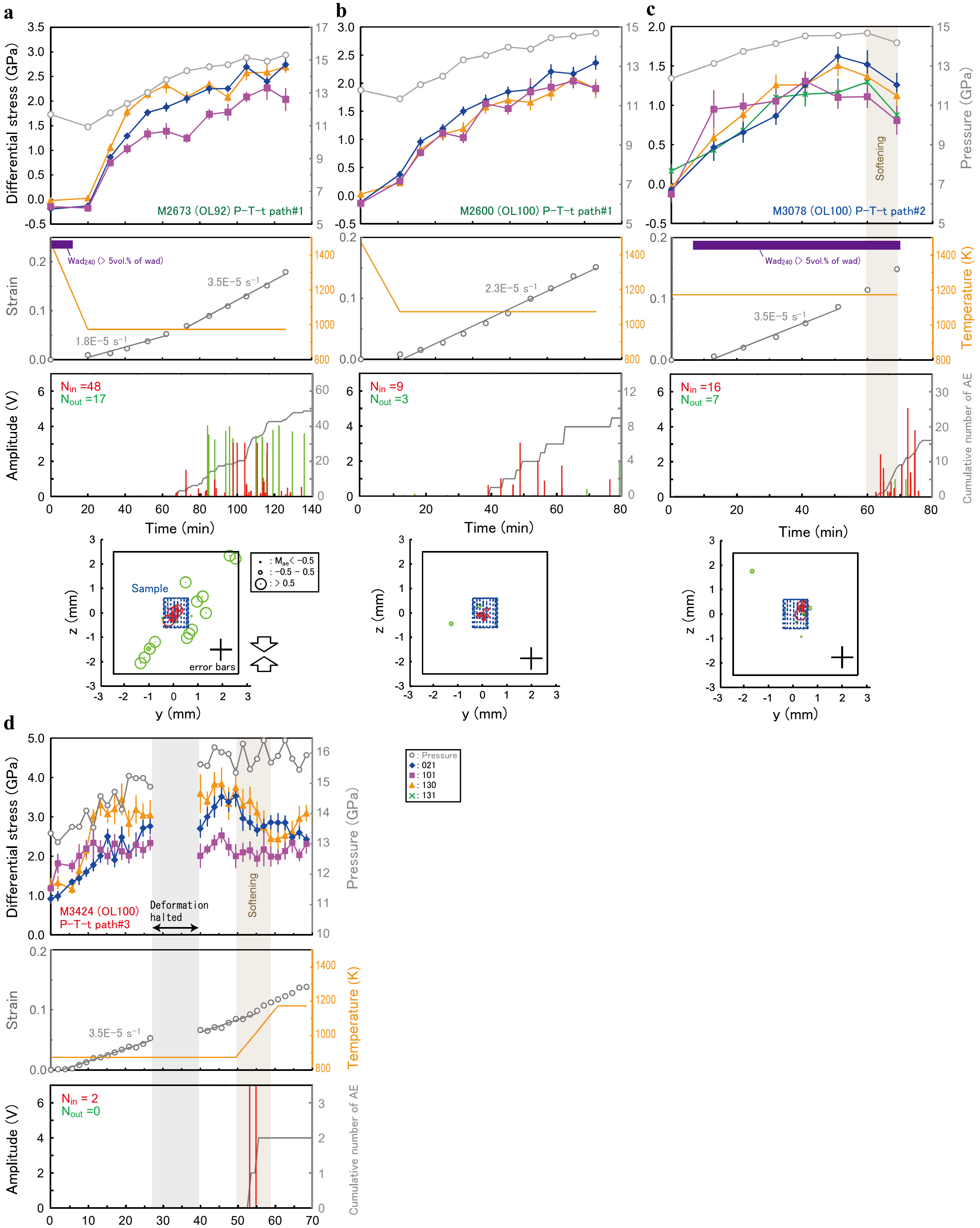

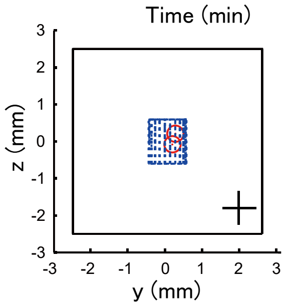

Supplementary Figure 2 | Mechanical (pressure, stress, and strain) and acoustic records plotted against time during the deformation stage in four runs in which strong acoustic emissions (AEs) were collected. (**a, b**) P-T-t path#1 (normal). (**c**) P-T-t path#2 (overpressurized just before the deformation). (**d**) P-T-t path#3 (temperature ramping during the deformation). Stress values were obtained from four diffraction peaks of olivine (solid diamonds: 021, solid squares: 101, solid triangles: 130, crosses: 131). Symbols, lines and error bars have the same meanings as shown in Fig. 3 and Supplementary Fig. 1.

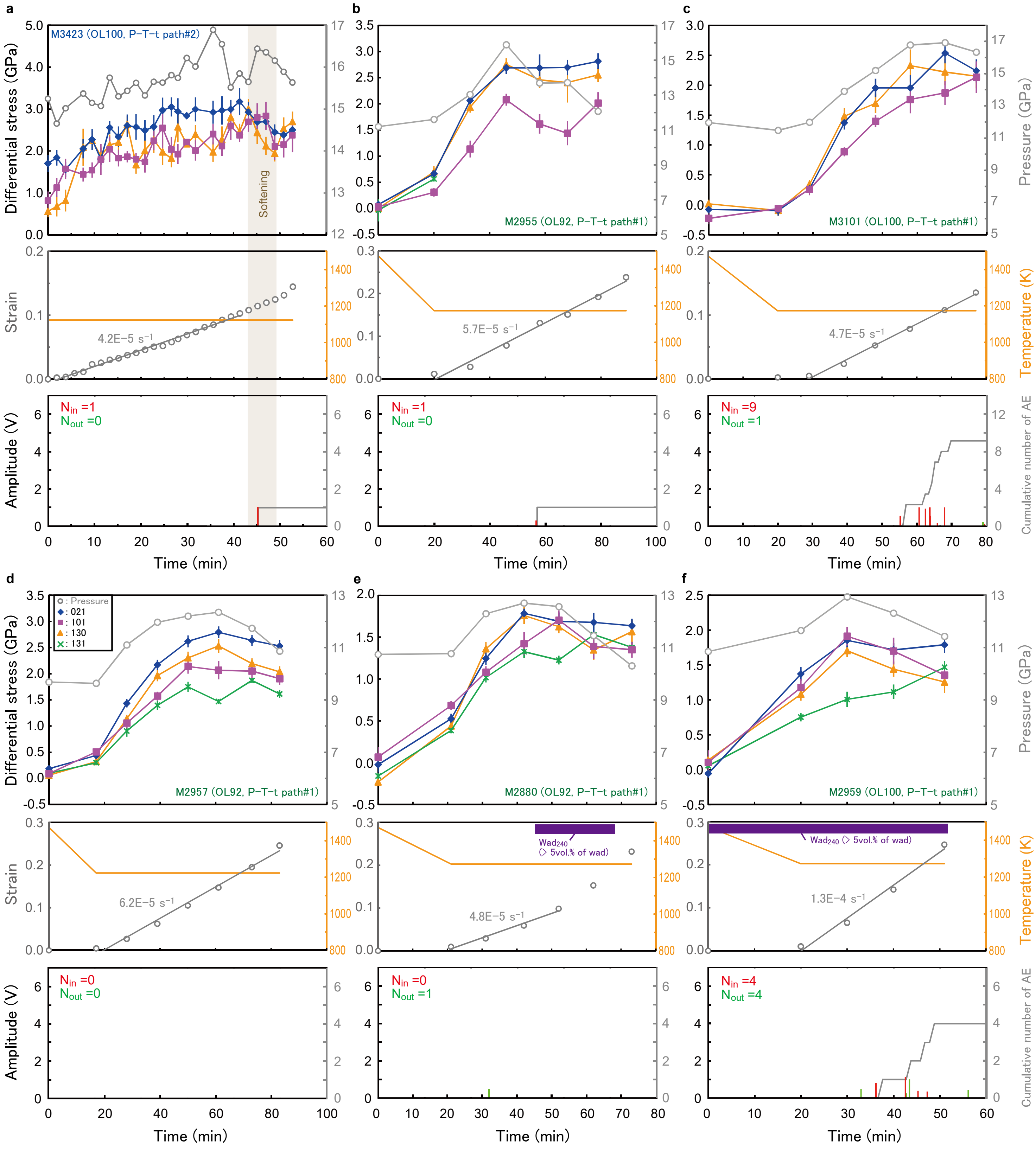

Supplementary Figure 3 | Mechanical (pressure, stress, and strain) and acoustic records plotted against time during the deformation stage in six runs in which acoustic activities were limited. (**a**) P-T-t path#2 (overpressurized just before the deformation). (**b-f**) P-T-t path#1 (normal). Stress values were obtained from four diffraction peaks of olivine (solid diamonds: 021, solid squares: 101, solid triangles: 130, crosses: 131). AE: acoustic emission. Symbols, lines and error bars have the same meanings as shown in Fig. 3 and Supplementary Fig. 1.

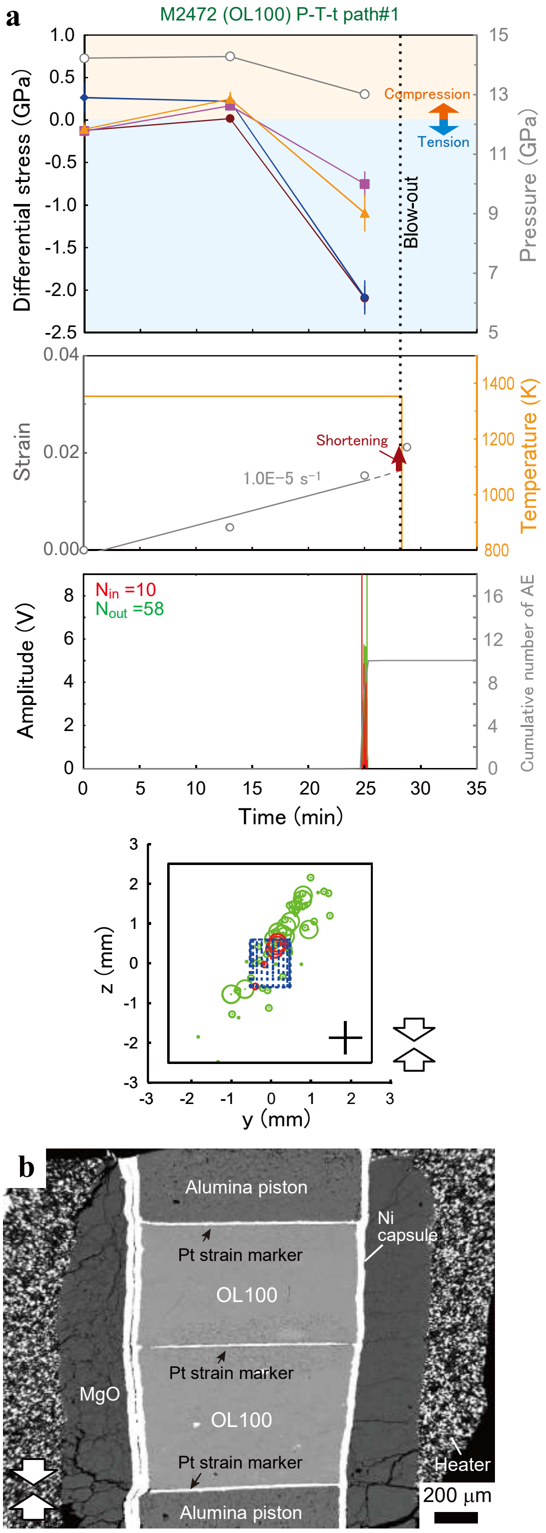

Supplementary Figure 4 | Mechanical (pressure, stress, and strain) and acoustic records from the deforming OL100 sample, which was suffered from a blow-out at 1350 K (M2472). (**a**) Mechanical data. Even though sample shortening proceeded during the deformation stage, strong tensile stresses (i.e., stress <0) associating intense acoustic emissions (AEs) was detected before the blow-out (25 min). Note that compressive stresses (i.e., stress >0) should be obtained in the uniaxial deformation geometry. A blow-out occurred at 28 min, resulting in a sudden shortening of the sample (the brown arrow). Symbols, lines and error bars have the same meanings as shown in Fig. 3 and Supplementary Fig. 1. (**b**) Backscattered electron image of the recovered sample. No throughgoing fault is observed.

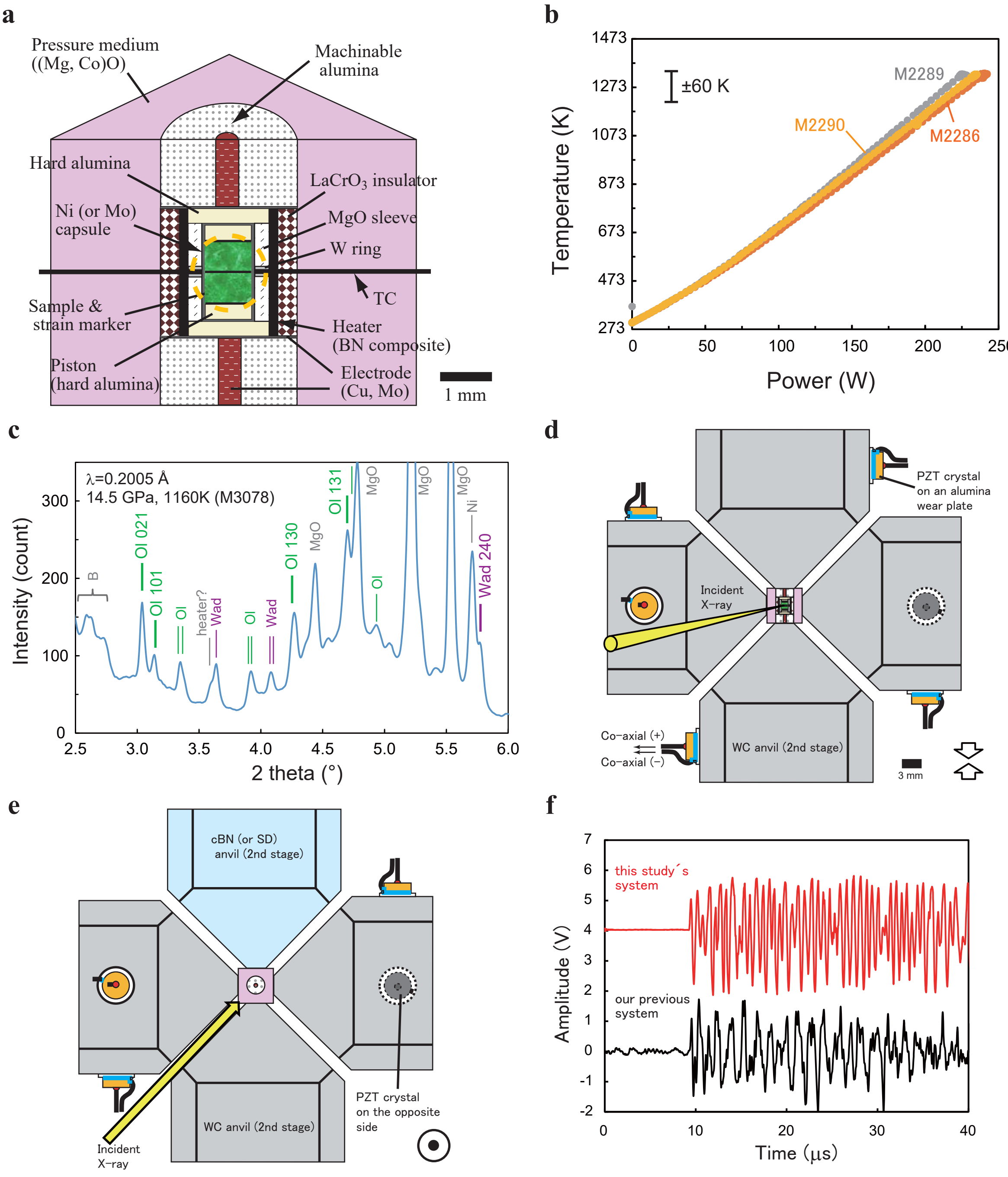

Supplementary Figure 5 | Experimental setup. (**a**) Cell assembly viewed in cross section from the direction parallel to the X-ray path (dashed orange circle). Note that the tungsten (W) rings and the thermocouple wires (TC) were not used for most of the deformation runs. (**b**) Calibration of central temperature in the cell vs. furnace power under 0.6 MN main-ram load (corresponding to ~13 GPa at 1250 K). Three calibration runs (M2286, M2289, and M2290) were conducted at the BL04B1 beamline, SPring-8. (**c**) One-dimensional diffraction pattern integrated from a half ring of the two-dimensional diffraction pattern taken at 14.5 GPa and 1160 K (M3078). Diffraction patterns of olivine (Ol), wadsleyite (Wad), the MgO sleeve, a nickel capsule (Ni) are observed. (**d, e**) Schematic representation of the experimental setup showing the positions of six PZT crystals (i.e., transducers) mounted on the sidewall surface of the second-stage anvils. Views from the directions perpendicular (**d**) and parallel (**e**) to the compressional direction. (**f**) Sample waveforms of an acoustic emission (AE) radiated from an OL100 sample deformed at ~15 GPa and 1160 K. Single AE event was monitored by two transducers: one was connected to a low-noise 30 dB pre-amplifier via an ultra-small pre-amplifiers (20 dB gain) (this study), and the other was connected to a low-noise 40 dB pre-amplifier used in our previous studies.

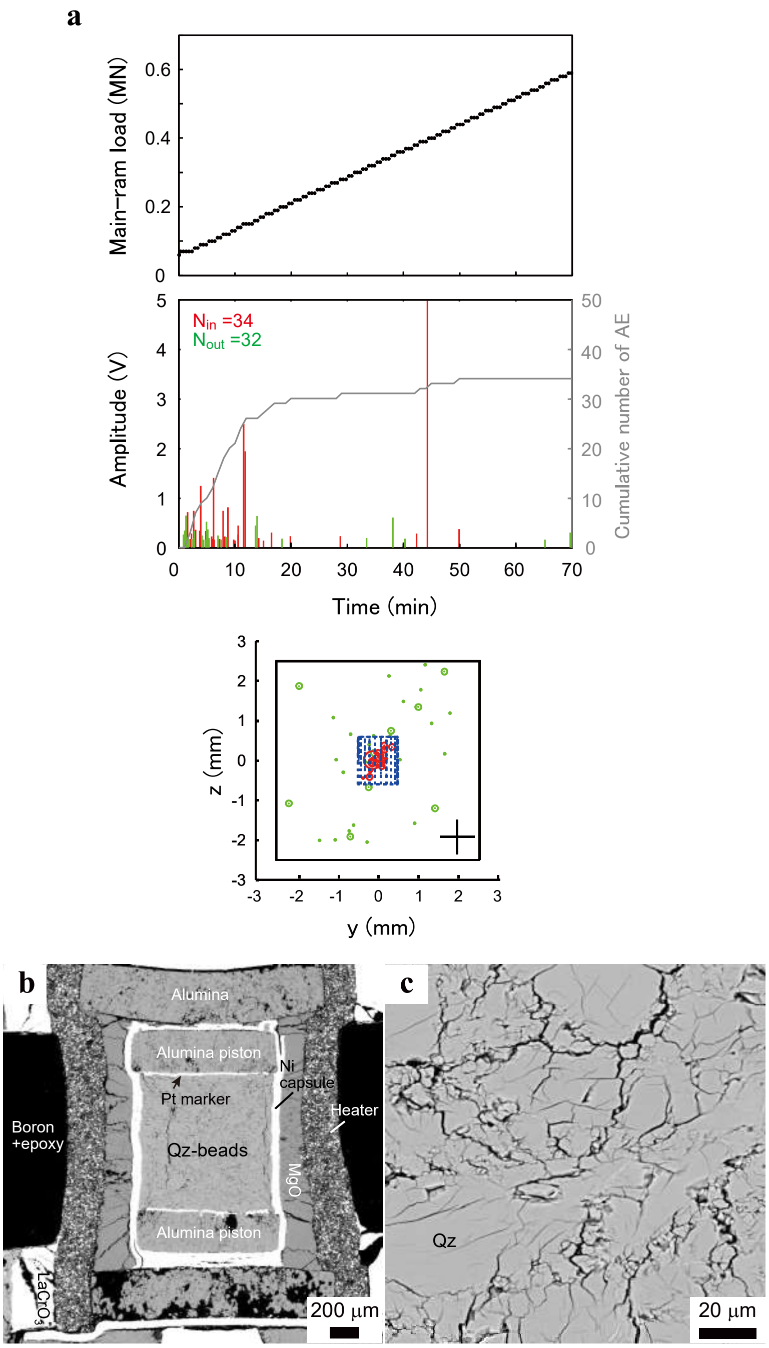

Supplementary Figure 6 | Acoustic records during cold compression of quartz beads (M0637). (**a**) Main-ram load and continuous acoustic records. Symbols and lines have the same meanings as shown in Fig. 3. (**b**) Backscattered electron image of the recovered cell assembly. (**c**) Magnified view of the quartz-beads sample. Qz: quartz. AE: acoustic emission.

Supplementary Table 1 | Flow law parameters for olivine and wadsleyite used for the calculations.

	n	r	p	A (m^p s^{-1} MPa^{-n-r})	E^* (kJ/mol)	V^* (cm^3/mol)	Source
Peierls creep of dry olivine	–––	–––	–––	$10^{12.1}$	502	30	Evans and Goetze (1979), Kawazoe et al. (2009)
Peierls creep of wet olivine	–––	–––	–––	$10^{17.3}$	502	30	Evans and Goetze (1979), Kawazoe et al. (2009), Ohuchi (2022)
Dislocation creep of wet olivine	3.5	1.2	0	1600	520	22	Hirth and Kohlstedt (2003)
dislGBS of olivine	3	1.25	1	$10^{-4.9}$	423	18	Ohuchi et al. (2015)
Superplasticity of forsterite	2.1	0	2	$10^{-7.6}$	302	4	McDonnel et al. (1998), Fei et al. (2016)

	$D_{0,l}$ (m^2/s)[a]	$\delta D_{0,gb}$ (m^3/s)[a]	H_l^* (kJ/mol)[a]	H_{gb}^* (kJ/mol)[a]	Ω (cm^3/mol)	Source
Diffusion creep of olivine	$10^{-5.9}$	$10^{-15.5}$	442 [b]	270	43.8	Fei et al. (2013, 2016)
Diffusion creep of wadsleyite	$10^{-7.6}$	$10^{-14.9}$	409	327	39.2	Shimojuku et al. (2009)

[a] Values for silicon diffusion.

[b] A calclated value for 13 GPa using the activation energy and the activation volume.
