# Supplementary material for: In situ X-ray and acoustic observations of deep seismic faulting upon phase transitions in olivine
Source: Nat Commun. 2022 Sep 15;13:5213. doi: 10.1038/s41467-022-32923-8 (PMC9477848; doi:10.1038/s41467-022-32923-8)
Supplement: Supplementary file 3 — Source Data [file 41467_2022_32923_MOESM3_ESM.pdf]

The raw X-ray data files (two-dimensional X-ray diffraction patterns and radiographic images) for each run are available from the links shown below:

http://earth.sci.ehime-u.ac.jp/~ohuchi/download/m2600.zip
http://earth.sci.ehime-u.ac.jp/~ohuchi/download/m2673.zip
http://earth.sci.ehime-u.ac.jp/~ohuchi/download/m2676.zip
http://earth.sci.ehime-u.ac.jp/~ohuchi/download/m2880.zip
http://earth.sci.ehime-u.ac.jp/~ohuchi/download/m2955.zip
http://earth.sci.ehime-u.ac.jp/~ohuchi/download/m2957.zip
http://earth.sci.ehime-u.ac.jp/~ohuchi/download/m2959.zip
http://earth.sci.ehime-u.ac.jp/~ohuchi/download/m3078.zip
http://earth.sci.ehime-u.ac.jp/~ohuchi/download/m3100.zip
http://earth.sci.ehime-u.ac.jp/~ohuchi/download/m3101.zip
http://earth.sci.ehime-u.ac.jp/~ohuchi/download/m3423.zip
http://earth.sci.ehime-u.ac.jp/~ohuchi/download/m3424.zip
http://earth.sci.ehime-u.ac.jp/~ohuchi/download/m3425.zip
http://earth.sci.ehime-u.ac.jp/~ohuchi/download/m3426.zip

Files of two-dimensional X-ray diffraction patterns are stored in the folder "run# & ADX & .zip" (e.g., m2600ADX.zip).

Files of radiographic images are stored in the folder "run# & radiograph & .zip" (e.g., m2600radiograph.zip).
